# 

**Published:** 1850-09

**Authors:** 


### *NOTICE TO SUBSCRIBERS.*

With this Number we send bills to our delinquent snbscribers. This will be enough to convince them that we want our money. The bills of old delinquents are made out at the original pricε, $2 per volume. This will be received if remitted free of expense, promptly ; but if we are under the necessity of employing collectors, which we shall do, the additional dollar for last year will be insisted upon.

---

### THE TRANSACTIONS OF THE ILLINOIS STATE MED. SOCIETY

Are now ready for delivery, and will, in a few days, be mailed to all members who have paid the assessment. Single copies may be obtained by enclosing 25 cents, posl-paid, to me, at Chicago, or six copies will be forwarded for one dollar.

E. G. MEEK,
*Sec'y Ills. State Med. Soc.*

### LIST OF RECEIPTS FOR JULY AND AUGUST, 1850.

| | |
|---|---|
| **ILLINOIS.** | |
| Drs. Samuel Thompson, | $2 00 |
| R. Rouse, | 2 00 |
| F. C. Hageman, | 4 00 |
| I. V. Goltra, | 2 00 |
| R. F. Adams, | 2 00 |
| E. Ingals, | 2 00 |
| H. Wasson, | 2 00 |
| S. Terwilliger, | 2 00 |
| J. C. Bardwell, | 2 00 |
| J. R. Snelling, | 3 00 |
| J. W. Freer, | 2 00 |
| Thos. Stanton, | 3 00 |
| C. W. Knott, | 2 00 |
| Geo. Higgins, | 2 00 |
| J. W. Spalding, | 2 00 |
| G. W. P. Harding, | 2 00 |
| E. S. Blanchard, | 2 00 |
| Dr. Smick, | 2 00 |
| Duffield & Wilhite, | 2 00 |
| D. W. C. Allen, | 4 00 |
| J. H. Gibson, | 4 00 |
| **WISCONSIN.** | |
| Drs. N. L. Gaston, | 4 00 |
| E. W. Gro, | 4 00 |
| C. H. B cknell, | 3 00 |
| A. P. Coryell, | 4 00 |
| E. Lewis, | 5 00 |
| Geo. H. Young, | 5 00 |
| E. R. Utter, | 3 00 |
| Drs. A. H. Bowe, | $1 00 |
| A. H. Vanorstrand, | 2 00 |
| E. Delaney, | 2 00 |
| Cutting Marsh, | 2 00 |
| Yomans & Ambler, | 2 00 |
| R. T. Goodwin, | 2 00 |
| W. Blackman, | 2 00 |
| Jno. W. Kimball, | 2 00 |
| N. C. Rowley, | 2 00 |
| **INDIANA.** | |
| Drs. Martin, | 2 00 |
| Allen Furnace, | 2 00 |
| Jefferson Miller, | 2 00 |
| James Wright, | 2 00 |
| W. Townsend, | 2 00 |
| W. M. Crowder, | 2 00 |
| T. L. Catherwood, | 75 |
| D. Hutchinson, | 2 00 |
| R. H. Sear, | 2 00 |
| W. C. Smyth, | 2 00 |
| **IOWA.** | |
| Drs. R. W. Hall, | 2 00 |
| C. W. Cowles, | 2 00 |
| **OHIO.** | |
| Dr. F. E. Bailey, | 1 00 |
| **MICHIGAN.** | |
| John W. Hall, | 2 00 |
| **NEW YORK.** | |
| R. B. Landon, | 1 00 |

### NOTICE TO READERS AND CORRESPONDENTS.

We have received original communications from Drs. W. Townsend, B. F. Mullen, F. Brady, and C. A. Hathwell.

We have received, through S. C. Griggs & Co., of Chicago, from Lea & Blanchard, Essays on Puerperal Fever and other Diseases peculiar to Women. By Fleetwood Churchill, M. D., M. R. Q. A.

General Therapeutics and Materia Medica. By Robley Dunglison, M. D., etc. 2 vols., octavo. Part 3d, Surgical Anatomy, by Joseph Maclise, Surgeon.

Also from J. Keen, Jr., & Brother, from Lea & Blanchard, Bennett on the Uterus, and Carpenter's Prize Essay on the Use and Abuse of Alcoholic Liquors.

From Lindsay & Blackiston: Reeses Medical Formulary, and Cazeaux' Midwifery; all of which will be noticed hereafter.

We have also received numerous pamphlets, reports, speeches, and circulars, many of which will receive attention as time and space permit.

Owing to the sickness of one of the Editors, and the absence of the other, many vexatious typographical errors occurred in our last No., for which an apology is due.

